# Impact of Crosslinking Agent on Sorption Properties of Molecularly Imprinted Polymers in Relation to Silver

**DOI:** 10.3390/polym17152055

**Published:** 2025-07-28

**Authors:** Laura Agibayeva, Yevgeniy Melnikov, Dilnaz Kubiyeva, Ruslan Kondaurov

**Affiliations:** 1 Faculty of Chemistry and Chemical Technology, Al-Farabi Kazakh National University, Al-Farabi Ave. 71, 050040 Almaty, Kazakhstan; laura.agibaeva@kaznu.edu.kz (L.A.); dilnazkubiyeva@gmail.com (D.K.); 2 Biochemical Engineering Department, International Engineering and Technological University, Al-Farabi Ave. 93a, 050060 Almaty, Kazakhstan; melnikov@kazetu.kz

**Keywords:** molecular imprinting, crosslinker, sorption, sorption degree, sorption capacity, silver

## Abstract

Molecularly imprinted polymers (MIPs) for silver sorption were synthesized using diethylene glycol dimethacrylate (DEGDMA) and divinylbenzene (DVB) as crosslinking agents. Synthesis was carried out using a ratio template: monomer: monomer: cross-linker = 1:2:2:8. The yield of obtained imprinting structures was 63.2% and 67.8% for MIP(DEGDMA) and MIP(DVB), respectively. The MIPs were analyzed by FTIR analysis, which showed the presence of characteristic peaks indicating the presence of monomers and crosslinkers in the MIP structure. According to the results of SEM analysis, the average cavity size for MIP(DEGDMA) is 0.81 ± 0.20 μm and for MIP(DVB) is 0.68 ± 0.23 μm in diameter. MIP(DEGDMA)’s sorption degree is 66.08%, and its sorption capacity is 3.31 g/g; MIP(DVB)’s sorption degree is 78.35%, and its sorption capacity is 3.92 g/g. The desorption degree is 69.85% for MIP(DEGDMA) and 69.52% for MIP(DVB). For analysis of sorption kinetics, the Radushkevich and Elovich kinetic models were applied.

## 1. Introduction

Noble metals (Au, Ag, Pt, Pd, Rh, etc.) play a key role in high-tech industries, the economy, and everyday life. Their importance is growing with the development of green energy, medicine, and electronics due to their unique properties—chemical resistance, electrical conductivity, and catalytic activity [1,2,3,4]. However, since noble metals are non-renewable, their natural reserves are finite. As a result, extracting noble metals from discarded industrial and electronic waste not only helps mitigate the scarcity of these valuable resources but also contributes to reducing environmental pollution. Nowadays, the trend of growing demand for recycled metals is observed, which requires innovative methods of their extraction and processing [5,6,7,8,9,10,11,12].

The special place from the noble metals is given to silver. The rising demand driven by renewable energy technologies and electronic devices is surpassing current silver production levels, leading to potential shortages. Silver remains a critical metal for electronics and microelectronics [13,14]; medicine and biotechnologies [15,16,17,18,19]; and energy and green technologies [20,21,22].

Sorption is an effective method for concentrating and extracting silver from dilute solutions (ore leaching, industrial waste, electronic waste). The method is based on the physical adsorption or chemical binding of Ag^+^ ions with the functional groups of the sorbent. Nowadays, the following sorbents for silver extraction can be named: activated coal; ion-exchange resins; inorganic sorbents; and biosorbents [23,24,25,26,27,28,29]. As an alternative to the above-mentioned sorbents, the application of molecularly imprinted polymers (MIPs) can be assumed.

MIPs are synthetic, tailor-made materials designed to selectively recognize and bind target molecules (templates), mimicking natural molecular recognition [30,31]. They offer high selectivity, stability, and reusability, making them superior to conventional sorbents in many applications [32,33]. Key applications of MIPs can be named as follows: metal recovery [34]; environmental remediation [35]; medicine and diagnostics [36]; sensors [37,38]; and food safety and analysis [39,40]. The advantages of MIPs over traditional sorbents are presented in Table 1 [41,42,43,44,45].

Molecularly imprinted polymers are created by forming a polymer around a target molecule using functional monomers and cross-linkers; once the template is removed, the resulting cavities enable selective rebinding of the target from complex mixtures [46]. There are several studies on developing MIPs based on various monomers and crosslinkers for the extraction of silver [47,48]. For example, a biopolymer chitosan was used as a raw material and glutaraldehyde as a crosslinker for obtaining MIP for selective removal and preconcentration of Ag(I) ions from aqueous solutions [49,50]. As for organic monomers, various acrylic and pyridine compounds were used. The most often used functional monomers among pyridine ones are 2-vinylpyridine (2-VP) and 4-vinylpyridine (4-VP). For example, in [51], ion-imprinted polymer (UA-DSPE-IIP) nanoparticles based on 2-VP were developed for the selective extraction of silver ions using ultrasonic-assisted dispersive solid-phase extraction. The binding properties of silver ion-imprinted polymers based on 4-VP were characterized by equilibrium and kinetic models [52]. 4-VP was used for obtaining magnetic ion imprinted polymers using functionalized magnetite (Fe_3_O_4_) nanoparticles [53] and by coating on the surface of magnetic graphene oxide [54] as a magnetic core. Another example of magnetic MIP using Fe_3_O_4_-SiO_2_-TiO_2_ nanoparticles as a magnetic support of adsorbent and Ag(I)-2,4-diamino-6-phenyl-1,3,5-triazine (DPT) complex as the template molecule was obtained based on the most often used acrylic monomer—methacrylic acid (MAA) [55]. The latter was also used for obtaining an Ag(I)-ion imprinted polymer with silver (Ag) dithizone complex as a template [56] and by the formation of 2-(4-hydroxypent-3-en-2-ylideneamine) phenol complex with silver ions [57]. The peculiarity of all these studies is the use of a single monomer for obtaining MIP. Therefore, our study was devoted to the preparation of MIPs based on two monomers—MAA and 4-VP.

Another peculiarity of these studies is the use of ethylene glycol dimethacrylate (EGDMA) as a crosslinker. The crosslinker is an important component in MIP synthesis, determining the structural stability, binding capacity, and selectivity of the polymer [58]. The common crosslinkers used in molecular imprinting are ethylene glycol dimethacrylate (EGDMA), divinylbenzene (DVB), diethylene glycol di-methacrylate (DEGDMA), N,N′-Methylenebisacrylamide (MBA), and others [59]. Its type and concentration directly affect the mechanical rigidity of the polymer network, the accessibility of imprinted cavities, and the rebinding kinetics of the target molecule [60,61]. Therefore, the aim of the work is to study the influence of the crosslinker type on the sorption properties of MIPs in relation to silver.

## 2. Materials and Methods

### 2.1. Materials

Silver nitrate with the composition of the main product ≥ 99.9%, provided by “Laborpharma” (Kazakhstan), was used as a template.

Methacrylic acid (MAA) produced by “Fluka” (Buchs, Switzerland) with a purity of ≥98% was used as a monomer.

4-vinylpyridine (4-VP) produced by “Sigma Aldrich” (Burlington, MA, USA) with a purity of ≥95% was used as a monomer.

Azobisisobutyronitrile (AIBN) in powder form, with the composition of the main product ≥ 95%, produced by “AK Scientific” (Union City, CA, USA), was used as an initiator without pre-treatment.

Di(ethylene glycol) dimethacrylate (DEGDMA) with a purity of ≥95% produced by “Sigma Aldrich” (USA) was used as a cross-linking agent.

Divinylbenzene (DVB) produced by “Sigma Aldrich” (USA) was used as a cross-linking agent (contains > 300 ppm of stabilizer).

2-hydroxyethyl cellulose (HEC), average Mv = 90,000, viscosity 100–180 cP, density 0.6 g/mL at 25 °C, produced by “Sigma Aldrich” (USA), was used as a stabilizer.

Toluene marked as “pure for analysis” and provided by “Skat” (Kazakhstan) was used as a porogen.

Nitric acid (96%) provided by “Laborpharma” (Kazakhstan) was used for the purification of synthesized copolymers.

Ethanol produced by “Talgar Spirtzavod” (Kazakhstan), with the composition of the main product ≥ 96%, was used for the purification of synthesized copolymers.

Dialysis membranes with a pore size of 12–14 kDa produced by “Medicell Membranes Ltd.” (London, England) were used for the purification of synthesized copolymers.

Argon was used to create an inert medium during synthesis.

### 2.2. MIPs Synthesis

**Figure 1:**
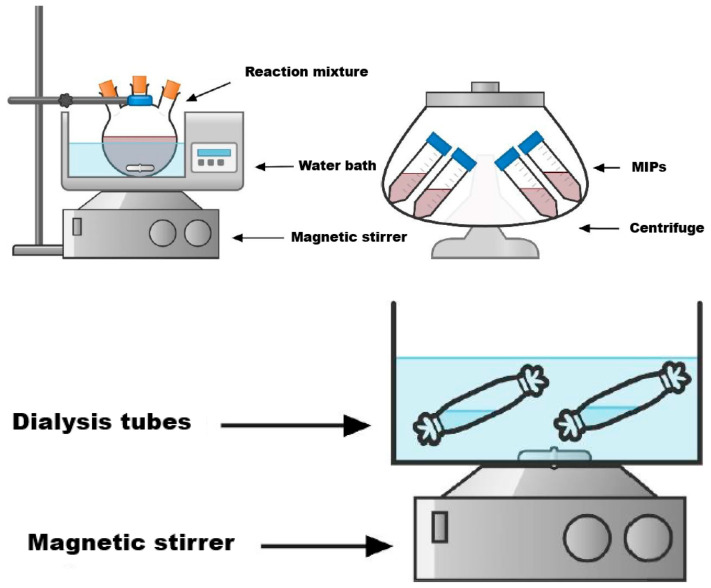
Synthesis of the MIPs.

The synthesis of molecularly imprinted polymer was carried out based on the methodology described in [62]. The synthesis of MIPs was carried out via bulk polymerization in the presence of two different cross-linkers: DEGDMA and DVB. A general representation of the MIP synthesis is shown in Figure 1.

Synthesis procedure sequence: template, two monomers, and crosslinker were taken in a ratio of 1:2:2:8. Firstly, 0.8493 g (5 mmol) of AgNO_3_, 0.848 mL (10 mmol) of MAA, 1.0769 mL (10 mmol) of 4-VP, and 40 mL of distilled water were mixed and stirred until the mixture was dissolved. Then, a 1% solution of HEC was prepared, 20 mL of which was added to the mixture along with 10 mL of toluene, and the mixture was stirred for 15 min. Afterwards, a definite amount of crosslinker (8.956 mL (40 mmol) of DEGDMA or 5.697 mL (40 mmol) of DVB) and 0.019 g of AIBN were added, and the mixture was stirred for 5 min. Inert argon was bubbled through the mixture in order to remove the oxygen. Then, the mixture was placed in a water bath at 60 °C for 6 h. The obtained product was put in a centrifuge to separate the thick part from the liquid part. After centrifugation, the thick part was purified by dialysis for 4 days: the first day in a solution of ethanol to remove unreacted monomers, the crosslinker, and porogen; the second day in distilled water to remove the unreacted monomers and HEC; the third day in a weak solution (0.5 M) of nitric acid to remove template molecules; and the last day again in distilled water to remove the remaining acid solution. Finally, the copolymer was freeze-dried. As a result, two MIP samples were obtained: MIP(DEGDMA) and MIP(DVB).

### 2.3. Physico-Chemical Analysis

*Freeze drying method* was used to dry the MIPs from water and obtain a powdered product using a Labconco 7934030 freeze dryer (Labconco Corporation, Kansas City, MO, USA). MIPs were first frozen at approximately −20 °C and then freeze-dried.

*The yield of the reaction product* is the ratio of the mass of the resulting product to the mass that should theoretically be obtained. The theoretical mass of the substances was taken as the total mass of the monomers used. The yield of products was calculated using the following formula:

(1) $$\omega =\frac{m_{pract.}}{m_{theoret.}}*100\%$$

*Fourier Transform Infrared (FTIR) Analysis* was conducted using an FTIR Spectrometer, Nicolet iS10 FT-IR Spectrometer (Thermo Electron Corporation, Waltham, MA, USA), for the determination of the MIP structure. Samples for analysis were prepared using the ATR technique.

*Scanning Electron Microscopy (SEM)* was conducted using a ZEISS Crossbeam 540 SEM (Carl Zeiss Microscopy GmbH, Jena, Germany) to identify the product’s surface morphology.

**Figure 2:**
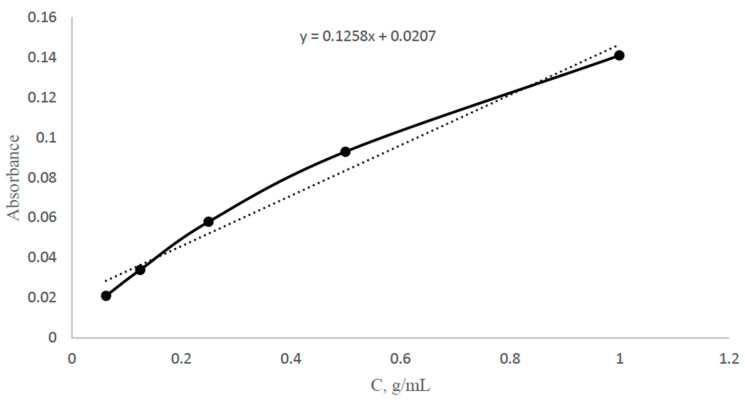
Calibration curve of silver nitrate at wavelength of 425 nm.

*Adsorption method* was used to study the sorption parameters (sorption degree, sorption capacity) of the imprinted structures. The optimal wavelength for a silver solution with a concentration of 0.5 g/100 mL vs. distilled water was obtained using a “Shimadzu UV-1900i” UV-VIS Spectrophotometer (Shimadzu, Kyoto, Japan). Then, 5 different concentrations of solutions (0.3125, 0.625, 1.25, 2.5, 5 g/L) were made, and a calibration curve for silver nitrate with a wavelength of 425 nm was constructed as shown in Figure 2. The sorption experiment was carried out as follows: each MIP (0.2 g) was placed in a solution of silver nitrate with a concentration of 0.1 g/mL. During sorption, the absorbance was measured on the spectrophotometer every 15 min for 3 h for further concentration calculations (using a calibration curve). After 24 h, control measurements were made. Based on the obtained results, the sorption degree and sorption capacity were calculated.

Sorption degree:

(2) $$\eta =\frac{C_{0}-C_{e}}{C_{0}}*100\%$$

where *C*_0_ is an initial concentration of silver nitrate in a solution, in g/mL, and *C_e_* is a concentration of silver nitrate in a solution at a certain time interval, in g/mL.

Sorption capacity:

(3) $$Q=\frac{\left(C_{0}-C_{e}\right)*V_{solution}}{m_{sorbent}}$$

where *C*_0_ is an initial concentration of silver nitrate in a solution, in g/mL; *C_e_* is a concentration of silver nitrate in a solution at a certain time interval, in g/mL; *V_solution_* is a volume of solution of terbium nitrate taken for sorption experiments, in mL; *m_sorbent_* is the mass of the MIPs taken for sorption experiments, in g.

*Desorption experiments* were conducted to study the polymer’s surface release properties. The samples of the imprinted structures after sorption were placed into beakers containing a weak solution of nitric acid at a concentration of 0.05 M for this purpose. Similar to the adsorption analysis, the absorbance was determined using a “Shimadzu UV-1900i” UV-VIS Spectrophotometer every 15 min for 3 h. A control absorbance measurement during desorption was performed at 24 h.

## 3. Results and Discussion

**Figure 3:**
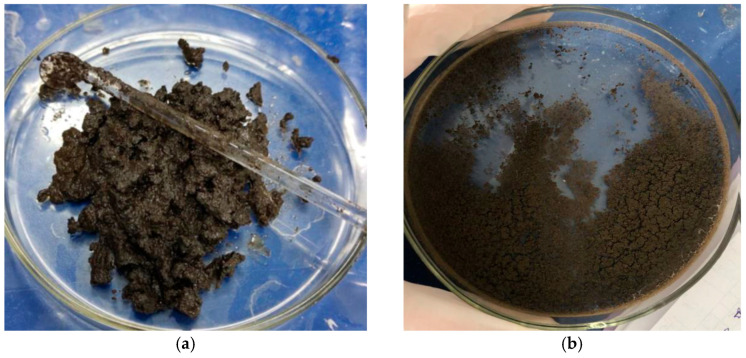
Synthesized MIPs: MIP(DEGDMA) (**a**) and MIP(DVB) (**b**).

The obtained MIPs are shown in Figure 3: MIP(DEGDMA) (a) and MIP(DVB) (b). Firstly, to analyze the obtained polymers, an organoleptic analysis was carried out (Table 2). The MIPs differ from each other in terms of appearance: the MIP(DVB) is dense while the MIP(DEGDMA) is more liquidized and looser. Due to the fact that 4-VP has a dark brown color, its presence in the MIPs also provides a dark color. The yield of the obtained imprinting structures was 63.2 and 67.8% for MIP(DEGDMA) and MIP(DVB), respectively. Thus, the yield of the synthesis product is around 65%, which is considered to be quite a productive result. The slight difference in values of the MIPs obtained with different crosslinkers can be explained by differences in the structure and reactivity (including steric factors) of the latter.

### 3.1. FTIR Analysis

**Figure 4:**
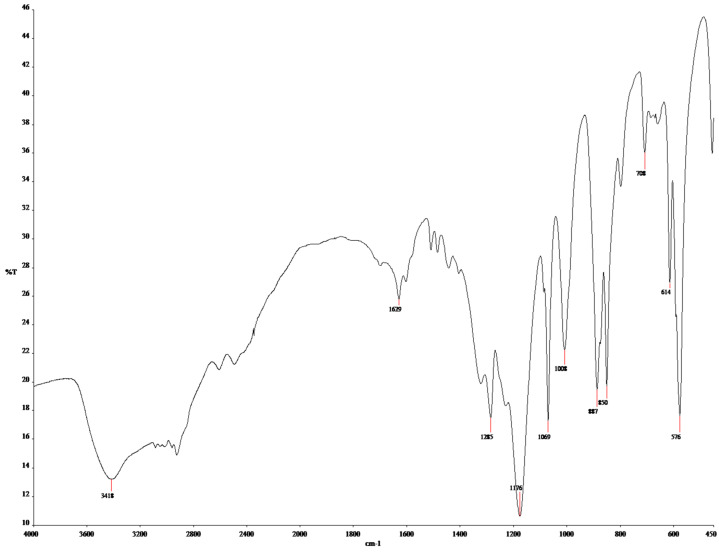
FTIR spectra of MIP(DEGDMA).

**Figure 5:**
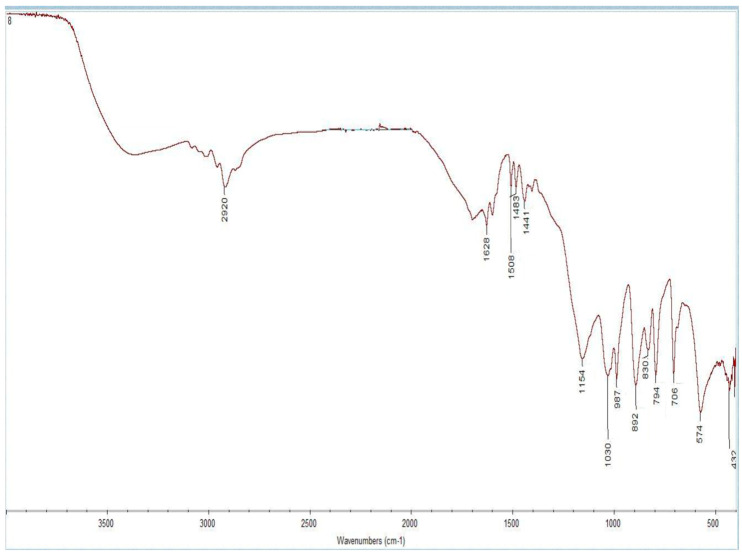
FTIR spectra of MIP(DVB).

The results of the FTIR spectroscopy are shown in Figure 4 for MIP(DEGDMA) and Figure 5 for MIP(DVB). The description of the peaks of the functional groups was analyzed according to the handbook in [63] and is shown in Table 3. As shown in Table 3, both monomers are present in structures on the obtained MIPs. The presence of methacrylic acid is shown by signals characterizing a carboxylic group: intra- and intermolecular hydrogen bonds between -OH groups at 3418.59 cm^−1^ for MIP(DEGDMA) and 3420 cm^−1^ for MIP(DVB); a -COOH group at 1629.57 cm^−1^ for MIP(DEGDMA) and 1628.91 cm^−1^ for MIP(DVB); an -OH in the carboxylic group at 887.15 cm^−1^ for MIP(DEGDMA) and 892,49 cm^−1^ for MIP(DVB). The presence of 4-vynilpyridine is characterized by signals of a pyridine ring at 1069.89 cm^−1^ for MIP(DEGDMA) and 1030.52 cm^−1^ for MIP(DVB). Also, both structures are characterized by a peak of -(CH_2_)_x_- at 708.02 cm^−1^ for MIP(DEGDMA) and 706.38 cm^−1^ for MIP(DVB), which shows that both monomers were polymerized during synthesis. The difference in the obtained MIPs is due to the crosslinkers used during synthesis, which have different structures. This was reflected in the signals observed in the FTIR spectra. The presence of DEGDMA is characterized by the following peaks: a -CH_2_-CO- group at 2920.06 and 1441.52 cm^−1^; a carbonyl group -C=O at 1285.25 cm^−1^; and a -C-O-C- group at 1176.37 cm^−1^. The structure of DVB in the obtained MIPs is shown by bands of variable intensity of the pyridine group at 1508.65 and 1483.93 cm^−1^ and by out-of-plane deformation vibrations of the 1,4-substitution at 794.67 cm^−1^. Thus, the results of the FTIR spectrometry show that the synthesis was successful.

### 3.2. Reaction Mechanism

**Figure 6:**
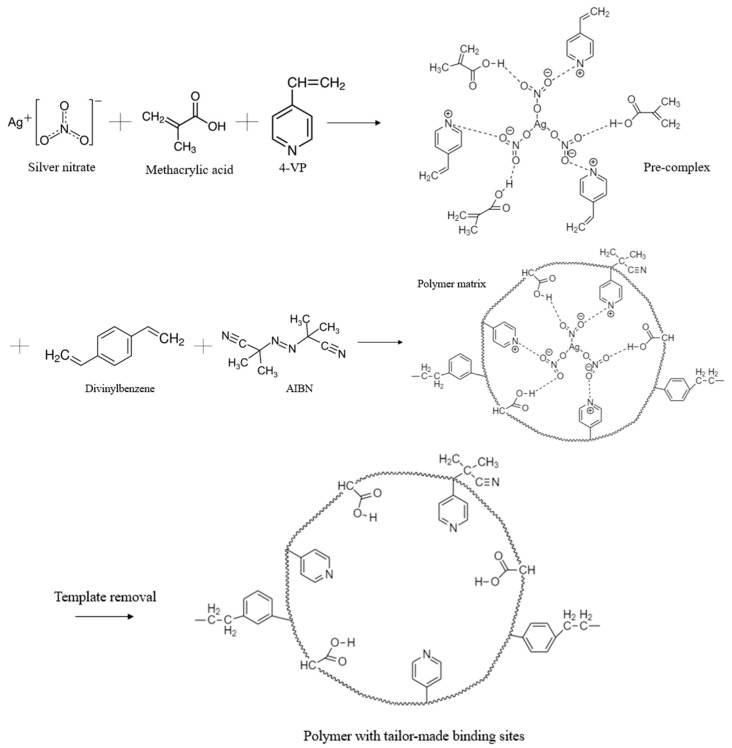
Proposed reaction mechanism for obtaining MIP(DVB).

The reaction mechanism for obtaining MIPs is proposed based on the literature [45,62] and the results of FTIR spectrometry, and is shown in the example of MIPs with DVB in Figure 6. The suggested mechanism firstly shows the formation of a pre-polymerization complex, in which the monomers and template are involved. Donor–acceptor bonds are formed between the pyridine group of the monomer 4-VP and template ions, and hydrogen bonds are formed between the monomer MAA and template molecules. Furthermore, the pre-polymerization complex is polymerized due to the disclosure of the double bonds of MAA and 4-VP. The polymerization process occurs in the presence of the initiator AIBN, which initiates the polymerization, and then, due to the presence of a crosslinker, the mesh structure is formed. Finally, during the process of dialysis, the template molecule is washed off or removed, and a polymer with molecular imprints is obtained. Molecular imprints are formed as complementary cavities.

### 3.3. SEM Analysis

**Figure 7:**
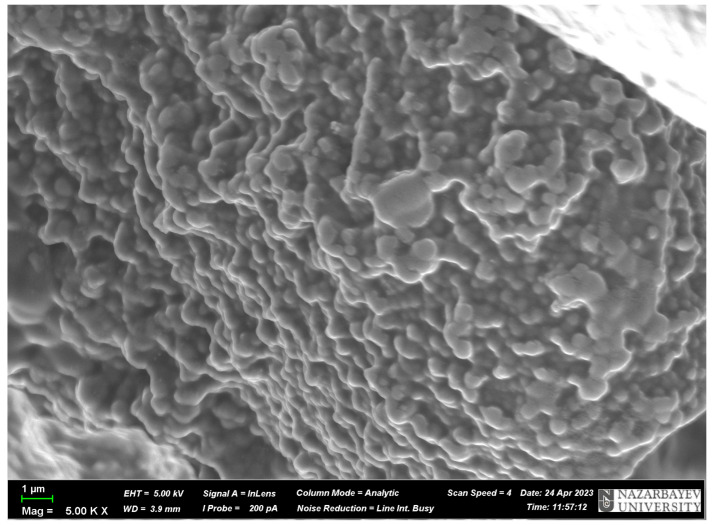
SEM image of MIP(DEGDMA).

**Figure 8:**
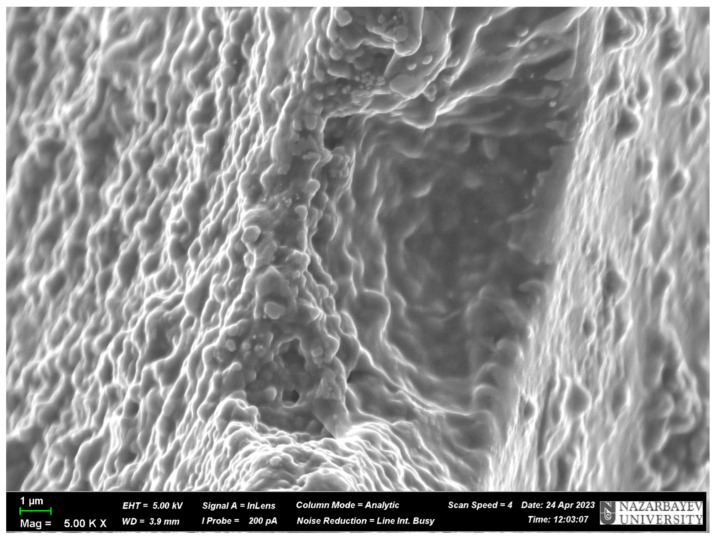
SEM image of MIP(DVB).

To prove the presence of the above-mentioned cavities, SEM analysis was carried out. The results of the SEM analysis are presented in Figure 7 and Figure 8. As can be seen from Figure 7 and Figure 8, the surface of the MIPs is not smooth, and they have some irregularities that look like cavities. Also, it can be seen that the structure of the MIPs is heterogeneous and dense, having in some places cavities due to the harsh formation of the monomeric structure, and these cavities are the molecular imprints, which were obtained in various forms and types. As the structure is heterogeneous, it can be concluded that the bond between the template and the monomer is not stable because the silver is ionizing, and perhaps it is present in an ionic form, which is not a stable bond. Also, the diameter of the cavities was measured using the program ImageJ (version 1.46r). As a result, the average cavity size for MIP(DEGDMA) is 0.81 ± 0.20 μm and for MIP(DVB) is 0.68 ± 0.23 μm in diameter. The difference in sizes of cavities could be explained by the difference in the structures of the crosslinkers: the DEGDMA molecule is more flexible due to its long chain structure, and DVB has an aromatic ring that can affect the steric factor.

### 3.4. Sorption Experiments

**Figure 9:**
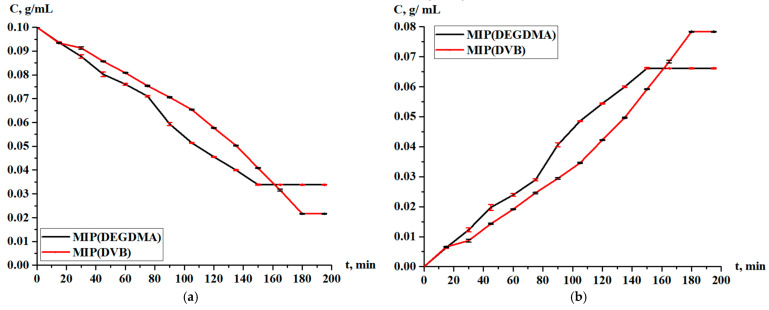
Concentration of silver nitrate during sorption in solution (**a**) and in the MIPs (**b**).

The obtained MIPs underwent a study of the sorption parameters in the case of the sorption of silver. Figure 9 shows the residual concentration of silver in the solution and the concentration of silver accumulated in the MIPs. According to the results, it is observed that sorption occurs throughout the entire experiment, and the curves lie too close to each other. After one hour, the MIP(DEGDMA) starts to sorb a slightly higher concentration of silver, but at 150 min, the value becomes constant, and its final concentration is less than is shown for MIP(DVB).

Based on the residual concentration data (Figure 9), the sorption parameters (sorption degree, sorption capacity) of MIP(DEGDMA) and MIP(DVB) were calculated.

**Figure 10:**
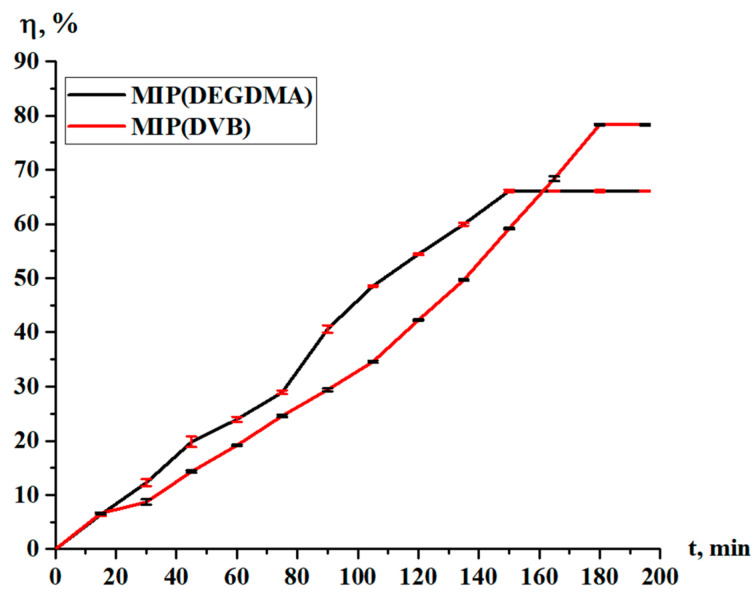
Sorption degree of the MIPs in relation to silver.

**Figure 11:**
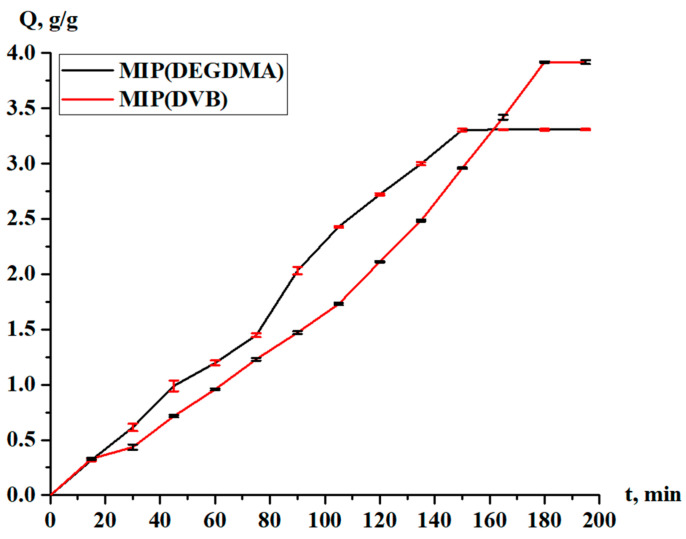
Sorption capacity of the MIPs in relation to silver.

The sorption degree and sorption capacity of the MIPs are shown in Figure 10 and Figure 11, respectively. The parameters increase with time, and more intense sorption of silver is observed in the case with MIP(DEGDMA). In the first 15 min, 6.39% of silver is sorbed by MIP(DEGDMA) and 6.62% of silver is sorbed by MIP(DVB). After that time, the characteristics of the sorption change, and MIP(DEGDMA) extracts more metal than MIP(DVB) up to 150 min (the difference is approximately 4–14%). The biggest difference is observed at 105 min: 48.56% vs. 34.59%. After 150 min, MIP(DEGDMA) reaches the equilibrium state, while MIP(DVB) continues the sorption of silver up to 180 min, after which equilibrium is reached. The values of the equilibrium sorption degree for MIP(DEGDMA) and MIP(DVB) are presented in Table 4, from which it is seen that after 180 min, equilibrium is reached (control measurements were obtained at 24 h). From the experimental data, it is seen that the sorption efficiency of MIP(DVB) is higher compared with MIP(DEGDMA). It can be supposed that MIP(DVB) has more stable cavities and higher binding capacity with silver molecules, while it has a looser structure. The same patterns are observed for the sorption capacity of MIPs. Also, it can be concluded that the saturation of MIP(DEGDMA) is faster than for MIP(DVB). Thus, the average sorption degree is approximately 72% and the average sorption capacity is 3.6 g/g, which is considered to be quite a productive result for MIPs.

### 3.5. Analysis of Sorption Kinetics

#### 3.5.1. Radushkevich Kinetic Model

The Radushkevich equation in the non-linear type is:

(4) $$q_{t}=q_{e}*(1-e^{-k_{R}*t})$$

where *q_e_* is the equilibrium sorption capacity (from the experimental data), in g/g; *q_t_* is an amount adsorbed at time *t*, in g/g; *k_R_* is a Radushkevich rate constant, in min^−1^; and *t* is the time, in min.

Linearization of the equation leads to the form:

(5) $$\ln\left(1-\frac{q_{t}}{q_{e}}\right)=-k_{R}*t$$

**Figure 12:**
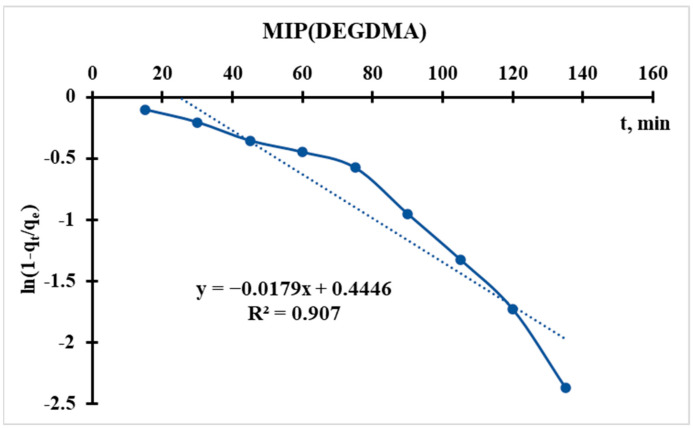
Radushkevich kinetic model’s curve for MIP(DEGDMA).

**Figure 13:**
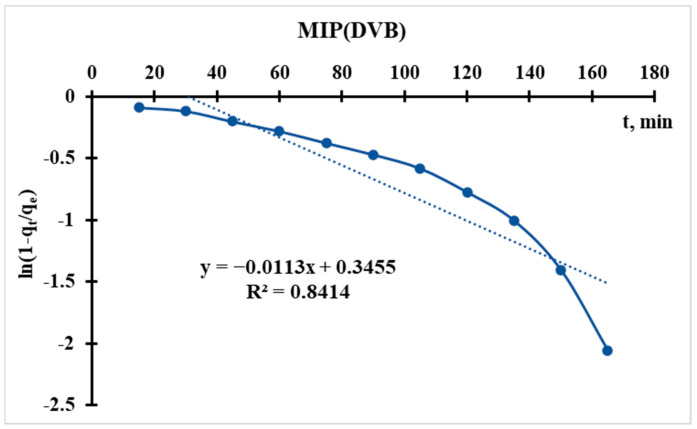
Radushkevich kinetic model’s curve for MIP(DVB).

Using the sorption data, the curve of ln(1 − q_t_/q_e_) = f(t) is plotted (Figure 12 and Figure 13), from which the following parameter is obtained:

(6) $$k_{R}=-Slope(S)$$

$$k_{R}(MIP(DEGDMA))=-\left(-0.0179\right)=0.0179\;{min}^{-1}$$

$$k_{R}(MIP(DVB))=-\left(-0.0113\right)=0.0113\;{min}^{-1}$$

In the Radushkevich model, a higher k_R_ indicates a faster sorption rate toward equilibrium. This suggests that MIP(DEGDMA) reaches equilibrium more quickly than MIP(DVB) under the same conditions.

The Radushkevich model is derived based on assumptions about the energy distribution in heterogeneous adsorption systems. A higher k_R_ implies that the adsorption process is more favorable or that the sorbent has a higher affinity for the adsorbate, leading to quicker saturation. MIP(DEGDMA) is likely more efficient in terms of kinetics, as it adsorbs the solute faster than MIP(DVB).

The difference in k_R_ values could arise from variations in:

- The cavity structure and surface area: MIP(DEGDMA) may have a more accessible cavity network or a higher surface area, facilitating faster diffusion and adsorption.
- The chemical affinity: MIP(DEGDMA) may have stronger interactions (e.g., electrostatic, van der Waals, or chemical bonding) with the adsorbate.
- The particle size: Smaller particles (if applicable) could lead to faster kinetics due to shorter diffusion paths.

Thus, according to the Radushkevich model, MIP(DEGDMA) saturates faster during silver sorption in comparison with MIP(DVB), but the latter sorbs a higher concentration of silver. These results correlate with the results of sorption experiments.

#### 3.5.2. Elovich Kinetic Model

The model is expressed as:

(7) $$q_{t}=\frac{1}{\beta }*ln\left(\alpha *\beta \right)+\frac{1}{\beta }*ln\left(t\right)$$

where *q_t_* is the sorption capacity at time t, in g/g; *α* is the initial sorption rate, in g·g^−1^·min^−1^; and *β* is the surface coverage parameter, in g/g (related to activation energy).

**Figure 14:**
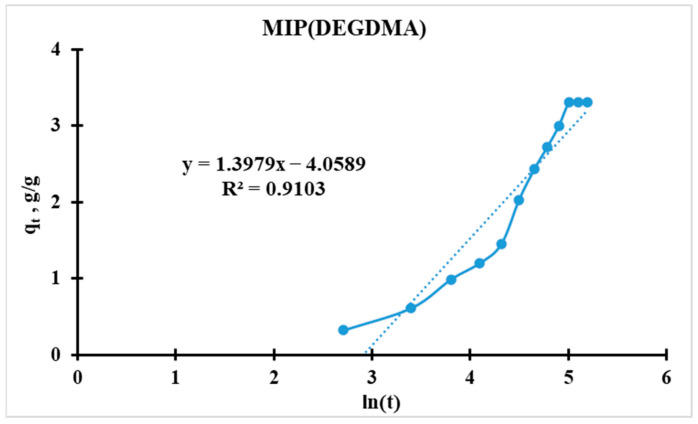
Elovich kinetic model’s curve for MIP(DEGDMA).

**Figure 15:**
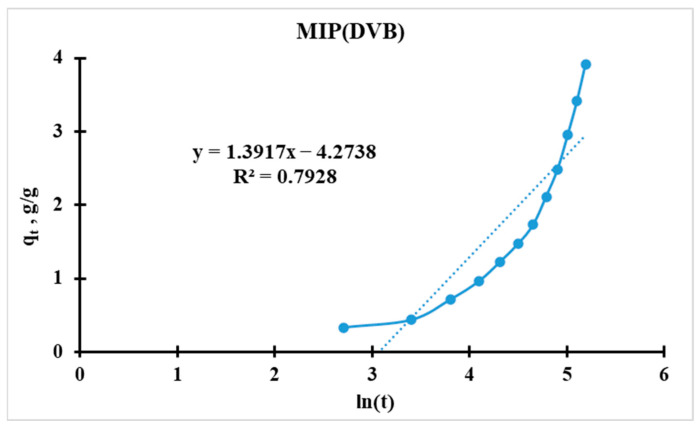
Elovich kinetic model’s curve for MIP(DVB).

For further analysis, the initial equation should be linearized (for plotting Figure 14 and Figure 15):

(8) $$q_{t}=a+b*ln(t)$$

Slope(*b*) = 1/*β*;

Intercept(*a*) = (1/*β*) ∗ ln(*α* ∗ *β*).

For the calculation of *α* and *β*, the following equations were used:

(9) $$\beta =\frac{1}{b}$$

(10) $$\alpha =\frac{e^{a*\beta }}{\beta }$$

$$\beta \left(MIP\left(DEGDMA\right)\right)=\frac{1}{1.3979}=0.7154\;g/g$$

$$\beta \left(MIP\left(DVB\right)\right)=\frac{1}{1.3917}=0.7185\;g/g$$

$$\alpha \left(MIP\left(DEGDMA\right)\right)=\frac{2.72^{\left(-4.0589*0.7154\right)}}{0.7154}=0.0172\;min^{-1}$$

$$\alpha \left(MIP\left(DVB\right)\right)=\frac{2.72^{\left(-4.2738*0.7185\right)}}{0.7185}=0.0139\;min^{-1}$$

Higher *α* indicates faster initial adsorption. MIP(DEGDMA) has a 24% higher initial rate than MIP(DVB) (0.0172 vs. 0.0139 min^−1^). MIP(DEGDMA) extracts silver more quickly at the early stages. These values correlate with the results calculated by the Radushkevich model.

Possible reasons for MIP(DEGDMA) higher α values:

- Higher porosity/microporosity: Faster diffusion of adsorbate into pores.
- More active surface sites: Functional groups (e.g., -OH, -COOH) accelerate initial binding.
- Smaller particle size: Larger surface area-to-volume ratio.

Thus, MIP(DEGDMA) saturates faster than MIP(DVB), which correlates with the results of the sorption experiments and the Radushkevich model.

Both sorbents have nearly identical β values (~0.715–0.719 g/g), suggesting similar binding energies and surface heterogeneity. Nearly identical β values imply:

- The same adsorption mechanism (e.g., chemisorption via ion exchange or complexation).
- Comparable surface heterogeneity (energy distribution of binding sites).

The surface coverage parameter of MIP(DVB) is slightly more than for MIP(DEGDMA). A higher β indicates stronger adsorbate–adsorbent interactions (chemisorption). These results correlate with the results of the sorption experiments, where it was proposed that the binding of MIP(DVB) with silver is stronger.

The results of both kinetic models (Radushkevich and Elovich) are similar and correlate with the results of the sorption experiments. The values of the sorption rate in both models are close to each other (Table 5); however, MIP(DEGDMA) has a higher sorption rate. Also, both kinetic models showed that MIP(DVB) sorbs a higher concentration of silver in comparison with MIP(DEGDMA).

Thus, there is a similarity in the sorption process of both MIPs, so the sorption is provided mainly by complexation with monomer units, but slight differences are influenced by the differences in the structures of the crosslinkers.

### 3.6. Desorption Experiments

For effective use of the synthesized MIPs, it is necessary to study their desorption properties. The process of desorption is closely connected with the regeneration procedure of the sorption parameters of the sorbents. Desorption was carried out with 0.05 M nitric acid.

**Figure 16:**
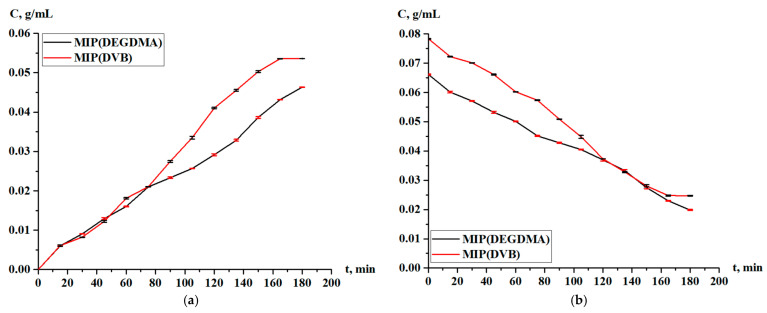
Concentration of silver nitrate during desorption in solution (**a**) and in the MIPs (**b**).

**Figure 17:**
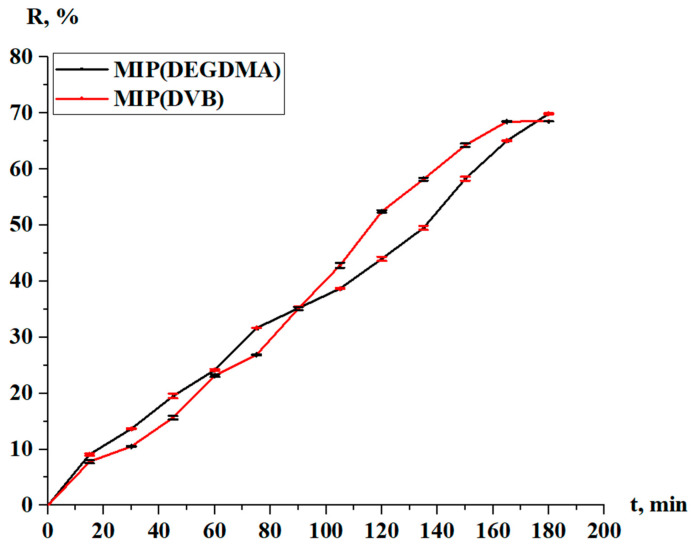
Desorption degree of the MIPs.

Figure 16 represents the concentration of silver in the solution and in the MIPs during desorption. Figure 17 shows the desorption degree of the MIPs. From the obtained experimental data, the following dependence is observed: in the first 90 min, the desorption rate of MIP(DEGDMA) is 1–5% greater in comparison with MIP(DVB), starting from 9.10% vs. 7.80% (15 min) and ending with 35.17% vs. 35.11% (90 min). After 90 min, the desorption of silver from the MIP(DEGDMA) matrix occurs more slowly, while desorption from MIP(DVB) becomes more rapid for 3–9%. Only at the end of the experiment (180 min), the final values of the desorption degree are slightly higher for MIP(DEGDMA). Control measurements were obtained after 24 h, and the results are presented in Table 6. The equilibrium value of the desorption degree at 24 h reaches almost 80%. Thus, it can be concluded that MIPs have quite high desorption efficiency, and they could be applicable as sorbents for silver recovery.

From the obtained results, it can be observed that the desorption degree of the MIP(DEGDMA) is slightly higher than that of the MIP(DVB). This can be explained by the binding properties of DVB and DEGDMA. The binding in the MIP(DVB) is stronger; thus, the molecules of the silver template are placed tightly in the cavities. The molecular chain of DEGDMA is longer than that of DVB; consequently, the structure of the MIPs will have some voids between the cavities. Because of that, the structure becomes less dense, and the molecules of the silver template will also be less dense, which leads to easier extraction of the silver template molecules from the copolymer matrix.

## 4. Conclusions

Based on the data obtained, the following conclusions can be made:

(1) The molecularly imprinted structures with silver salt as the template were synthesized with the ratio of template:monomer:monomer:cross-linker = 1:2:2:8. The obtained imprinted structures have a dark brown color due to the presence of the monomer 4-VP. MIP(DVB) has a dense structure, whereas MIP(DEGDMA) has a looser structure.
(2) The structure of the MIPs is dense and heterogeneous, with molecular imprint cavities formed due to unstable bonding between the template and the monomer, likely influenced by the ionized state of silver. The average cavity sizes were 0.81 ± 0.20 μm for MIP(DEGDMA) and 0.68 ± 0.23 μm for MIP(DVB), with the difference attributed to the flexibility of DEGDMA’s long-chain structure compared to DVB’s rigid aromatic ring.
(3) A mechanism of chemical reaction for obtaining a molecularly imprinted system with a silver template was suggested, which includes the formation of a pre-polymerization complex, the polymerization of monomers, and cross-linker around a template molecule. In the formation of a pre-polymerization complex, it is supposed that donor–acceptor bonds are formed between the pyridine group of the monomer 4-vinylpyridine and template ions, and hydrogen bonds are formed between the monomer methacrylic acid and template molecules.
(4) MIP(DEGDMA) saturates faster during silver sorption in comparison with MIP(DVB), but the latter sorbs a higher concentration of silver. The average sorption degree is approximately 72% and the average sorption capacity is 3.6 g/g, which is considered to be quite a productive result for MIPs.
(5) The sorption process was described using kinetic models (Radushkevich and Elovich). Both kinetic models showed identical results that correlated with the results of the sorption experiments: MIP(DEGDMA) saturates faster during silver sorption in comparison with MIP(DVB), but the latter sorbs a higher concentration of silver.
(6) The desorption degree reaches nearly 80% after 24 h, indicating that the MIPs possess high desorption efficiency and may be effectively used as sorbents for silver recovery.

Based on the results mentioned above, it can be concluded that the synthesized MIPs are promising as sorbents for the selective extraction of silver. Both MIPs exhibit similar sorption behavior, primarily driven by complexation with monomer units. However, minor variations arise due to structural differences in the crosslinkers.

## Figures and Tables

**Table 1:** Advantages of MIPs over traditional sorbents.

Feature	MIPs	Conventional Sorbents (e.g., Activated Carbon, Resins)
Selectivity	Extremely high (mimics antibodies)	Low to moderate
Stability	Resists harsh pH/temperature	Degrades under extreme conditions
Reusability	100+ cycles with minimal loss	Limited regeneration cycles
Customization	Designed for any target	Limited functional groups
Cost	Higher initial cost	Lower cost but less efficient

**Table 2:** Organoleptic analysis of obtained MIPs.

Properties	MIP(DEGDMA)	MIP(DVB)
Color	Dark brown	Dark brown
Density	Loose	Dense
Odor	Strong smell of monomer	Gasoline-like smell

**Table 3:** Comparison of FTIR spectra results for MIP(DEGDMA) and for MIP(DVB).

Functional Group	Wavenumber, cm^−1^ for MIP(DEGDMA)	Wavenumber, cm^−1^ for MIP(DVB)
-OH (inter- and intramolecular -H bonds in polymers)	3418.59	3420
-CH_2_-CO-	2920.06; 1441.52	-
-COOH	1629.57	1628.91
Aromatic ring	-	1508.65; 1483.93
-C=O	1285.25	-
-C-O-C-	1176.37	-
Pyridine ring	1069.89	1030.52
-OH in -COOH	887.15	892.49
1,4-substitution in aromatic ring	-	794.67
--(CH_2_)_x_-	708.02	706.38

**Table 4:** Sorption parameters of MIP(DEGDMA) and MIP(DVB) after 24 h.

Parameter	MIP(DEGDMA)	MIP(DVB)
Sorption degree, %	66.08 ± 0.26	78.35 ± 0.14
Sorption capacity, g/g	3.31 ± 0.01	3.92 ± 0.01

**Table 5:** Comparison of sorption rates for MIPs using kinetic models.

Kinetic Model	Sorption Rate, min^−1^	
	MIP(DEGDMA)	MIP(DVB)
Radushkevich	0.0179	0.0113
Elovich	0.0172	0.0139

**Table 6:** Desorption degree of MIP(DEGDMA) and MIP(DVB) after 24 h.

Parameter	MIP(DEGDMA)	MIP(DVB)
Desorption degree, %	79.85 ± 0.09	78.52 ± 0.01

## Data Availability

The original contributions presented in this study are included in the article. Further inquiries can be directed to the corresponding author.

## Abbreviations

The following abbreviations are used in this manuscript:

MIP	Molecularly imprinted polymer
MAA	Methacrylic acid
4-VP	4-vinylpyridine
DEGDMA	Diethylene glycol dimethacrylate
DVB	Divinylbenzene
AIBN	Azobisisobutyronitrile
HEC	Hydroxyethyl cellulose
FTIR	Fourier transform infrared spectroscopy
SEM	Scanning electron microscopy
